# Assessing Indoor Environmental Control Practices by Race/Ethnicity Among Children With Asthma in 14 US States and Puerto Rico, 2013–2014

**DOI:** 10.5888/pcd16.190199

**Published:** 2019-12-26

**Authors:** Faye M. Rozwadowski, Ginger L. Chew, Hatice S. Zahran, Melissa L. Santorelli

**Affiliations:** 1 Epidemic Intelligence Service, Division of Scientific Education and Professional Development, Centers for Disease Control and Prevention, Atlanta, Georgia; 2 National Center for Environmental Health, Asthma and Community Health Branch, Centers for Disease Control and Prevention, Atlanta, Georgia; 3 New Jersey Department of Health, Community Health and Wellness Unit, Trenton, New Jersey (formerly)

## Abstract

**Introduction:**

In the United States, children in Puerto Rico and non-Hispanic black children in the mainland US have a higher burden of asthma than non-Hispanic white children in the mainland US. We examined indoor environmental control (IEC) practices that reduce asthma triggers, by race/ethnicity among children in the mainland US and Puerto Rico.

**Methods:**

We used 2013 and 2014 data from the Behavioral Risk Factor Surveillance System Asthma Call-back Survey Child Questionnaire from 14 states and Puerto Rico to measure the association between race/ethnicity and IEC practices, adjusting for sociodemographic covariates, among children identified as ever receiving an asthma diagnosis. Racial/ethnic groups were compared in 14 US states using aggregated data. Separate analyses compared IEC practices for children diagnosed with asthma in Puerto Rico with children of all races/ethnicities diagnosed with asthma in 14 states.

**Results:**

Among households in 14 US states that had a child with asthma, non-Hispanic black children were more likely than non-Hispanic white children to use an air purifier (36.8% vs 25.2%; adjusted odds ratio [aOR] = 2.0; 95% confidence interval [CI], 1.3–3.2) and avoid pets in the bedroom (87.9% vs 58.3%; aOR = 4.5; 95% CI, 2.3–8.8). Children in Puerto Rico were more likely than children in 14 states to use dust mite–impermeable pillow covers (53.7% vs 36.4%; aOR = 3.6; 95% CI, 1.8–7.1) and mattress encasements (60.3% vs 30.3%; aOR = 2.4; 95% CI, 1.2–4.8).

**Conclusion:**

IEC practices such as using air purifiers, pillow covers, mattress encasements, and avoiding pets in the bedroom vary by race/ethnicity among children with asthma. These findings show that vulnerable populations are using IEC practices, but asthma prevention and control measures should continue to be assessed.

**Summary**

**What is already known on this topic?**

Indoor environmental control practices reduce asthma symptoms among children. Non-Hispanic black children and children in Puerto Rico are affected by asthma more than are non-Hispanic white children.

**What is added by this report?**

Households with non-Hispanic black children diagnosed with asthma may be more likely to use indoor environmental control (IEC) practices than households with non-Hispanic white children diagnosed with asthma. Households with children diagnosed with asthma in Puerto Rico may be more likely to use some IEC practices than children diagnosed with asthma living in the US mainland.

**What are the implications for public health practice?**

Observed differences in IEC practices demonstrate the need for clinicians to focus assessments and education efforts in a more targeted and customized manner for vulnerable pediatric patients.

## Introduction

Asthma is the leading chronic disease among children in the United States, the top reason for missed school days, and the third leading cause of pediatric hospitalizations (1). Racial/ethnic disparities exist in childhood asthma prevalence. In the United States, non-Hispanic black children are twice as likely as non-Hispanic white children to have received a diagnosis of asthma, 2 to 3 times as likely to be hospitalized for asthma, and 4 times as likely to die from asthma (2).

Puerto Rican children are an additional vulnerable population who experience the highest prevalence of asthma (11%) compared with non-Hispanic black (7.7%) and non-Hispanic white children (5.3%) in the US mainland. In Puerto Rico, 32% of children have received a diagnosis of asthma, compared with 12.6% of children in the US mainland (3). The lifetime odds of having received an asthma diagnosis among Puerto Rico island resident children born to Puerto Rico island residents is 2.5 times higher than United States mainland-born non-Hispanic white children with United States mainland-born parents (4).

The National Heart, Lung, and Blood Institute’s (NHLBI’s) Guidelines for the Diagnosis and Management of Asthma outline multiple indoor environmental control (IEC) practices to reduce asthma triggers. IEC practices in households of children with asthma have been shown to reduce asthma symptoms (5). A common asthma trigger, dust mites and their allergens, can be effectively controlled by washing bedding weekly in hot water, using dust mite–impermeable pillow covers and mattress encasements, and reducing household humidity levels. Preventing smoking in the home and keeping pets out of children’s bedrooms are additional recommended IEC practices.

Understanding racial/ethnic differences in the use of environmental control measures can help implement evidence-based interventions. The only comparable study to our assessment comes from the 2003 National Asthma Survey. This survey suggested that the use of mattress encasements and dust mite–impermeable pillow encasements is lower among non-Hispanic black and Hispanic children with asthma, compared with non-Hispanic white children with asthma. Nonetheless, the sampling frame included only 4 states and excluded participants with cellular telephones and residents of Puerto Rico (6). In addition, IEC practices were not examined separately among any of the subsets of Hispanic (eg, Puerto Rican or Mexican ethnicity) children in the reference study, which is a limitation given the disproportionate burden of asthma for children in Puerto Rico.

We assessed disparities in IEC practices by race/ethnicity among children with asthma using a large and inclusive sample from the Behavioral Risk Factor Surveillance System (BRFSS) Asthma Call-back Survey (ACBS).

## Methods

We performed a cross-sectional analysis using publicly available data from the 2013 and 2014 National BRFSS ACBS Child Questionnaire.

*Data source.* ACBS is an extension of BRFSS, a key surveillance system designed by the Centers for Disease Control and Prevention (CDC) to monitor modifiable health behaviors and conditions in all US states and territories using telephone surveys. Respondents to the BRFSS include noninstitutionalized, US civilian residents aged ≥18 years. The BRFSS ACBS is an ongoing, state-based and US territory landline and cellular telephone survey administered by BRFSS and developed and funded by the CDC National Asthma Control Program, Asthma and Community Health Branch, in the CDC National Center for Environmental Health. States and US territories included in this assessment met CDC data quality standards (at least 6 months of data collection and at least 75 records) and participated in the landline and cellular phone child ACBS for either one or both years (ie, 2013 and 2014). For 2013, Connecticut, Indiana, Michigan, Mississippi, Montana, Nebraska, New Jersey, Texas, Utah, and Washington were included. For 2014, Georgia, Indiana, Kentucky, Maryland, Michigan, Nebraska, New Jersey, Pennsylvania, Texas, Utah, and Puerto Rico were included. Annual sample weights published by CDC were rescaled to account for the number of years and varying sample sizes per year per state and US territory (7). ACBS is conducted approximately 2 weeks after the initial BRFSS is completed. If the respondent from a randomly selected household on the BRFSS answers that they have a child (aged <18 years) who has received an asthma diagnosis, then that household is eligible for the in-depth child ACBS. The response rate for households that completed the ACBS Child Questionnaire for 2013 was 44.6% (8) and for 2014 was 46.3% (9). Annual survey data published by CDC include survey design variables that were used for the analysis. Additional details about survey methods and the recommended use of these design variables are available (7).

*Study population*. Our study population included households with children identified on BRFSS by the adult respondents as having a diagnosis of asthma and answering questions on the ACBS (N = 1,478).

*Outcome*. The outcome of interest was the use of IEC practices in households of children with asthma. The BRFSS ACBS asks adult respondents multiple IEC questions. These questions include whether an air cleaner/purifier or a dehumidifier is regularly used inside the child’s home, whether an exhaust fan venting to the outside is used regularly when cooking in the kitchen, use of an exhaust fan in a child’s bathroom that vents to the outside or a pillow [or mattress] cover that is made especially for controlling dust mites, whether the child’s sheets and pillowcases are washed in hot water, whether a pet is allowed in the child’s bedroom, and whether anyone has smoked inside the child’s home in the past week.

*Race/ethnicity and other variables.* Race/ethnicity of the child was imputed from answers provided by adults about their respective child on the originating BRFSS. Children from the 14 states included were classified as non-Hispanic white; non-Hispanic black; Puerto Rican; other Hispanic; and non-Hispanic other race. Children of households in Puerto Rico were categorized as Puerto Rico Island residents. Other study variables included adult respondent’s education level (ie, did not graduate high school, graduated high school, attended college or technical school, or graduated from college or technical school), child’s age (0–4 years, 5–9 years, 10–14 years, and 15–17 years), and child’s sex.

*Statistical analyses*. We analyzed the data using SAS version 9.4 (SAS Institute, Inc) procedures that accounted for the complex survey design. We examined the percentage of households of children with asthma that implement IEC practices by race/ethnicity. We used the Rao-Scott χ^2^ test to measure the unadjusted bivariate association between race/ethnicity and IEC practices. We used logistic regression to assess the association between race/ethnicity and use of IEC practices among children with asthma, adjusted for age, household income, and parental education level. We used separate models to 1) compare children from the continental states by race/ethnicity, and 2) compare children from Puerto Rico to children of the 14 US mainland states aggregated. Significance was set at *P* < .05.

## Results

Children aged 5–14 years were the largest represented age groups from which data were drawn for the 14 states and Puerto Rico (Table 1). Almost 30% of children from the 14 states were from a household with an annual income <$25,000; 49% had a parent or legal guardian without a college degree; and approximately half were of a racial/ethnic minority. Sixty-one percent of the children in Puerto Rico were from a household with an annual income <$25,000, and more than half had a parent or legal guardian without a college degree.

**Table 1:** Characteristics of Children With Diagnosis of Asthma, Behavioral Risk Factor Surveillance System, Child Asthma Call-Back Survey, 14 US States^a^ and Puerto Rico, 2013–2014

Characteristics	14 US States (n = 1,418)	Puerto Rico (n = 60)
	Estimated % (95% Confidence Interval)	
**Age, y**		
0–4	15.6 (12.2–19.0)	19.7^b^ (6.7–32.6)
5–9	31.8 (27.7–35.9)	43.8 (28.4–59.3)
10–14	33.1 (29.2–36.9)	25.5 (14.0–37.0)
15–17	19.5 (16.1–22.9)	11.0^b^ (3.4–18.6)
**Household income, $**		
<25,000	27.6 (24.2–31.0)	60.8 (46.6–75.0)
25,000–49,999	16.6 (13.3–19.9)	24.7 (12.3–37.0)
50,000–74,999	10.9 (8.4–13.4)	c
≥75,000	37.3 (33.3–41.4)	c
Refused or unknown	7.5 (4.9–10.2)	9.3^b^ (0.95–17.6)
**Education level of parent**		
No high school diploma	8.1 (6.1–10.2)	c
High school graduate	16.8 (14.0–19.5)	34.7 (20.3–49.2)
Some college or technical school	24.3 (21.4–27.3)	21.1 (9.3–33.0)
College or technical school graduate	43.3 (39.3–47.3)	39.2 (25.1–53.4)
Refused/unknown	7.5 (5.2–9.7)	c
**Race/ethnicity**		
Non-Hispanic white	53.3 (49.0–57.7)	NA
Non-Hispanic black	22.0 (18.2–25.8)	NA
Puerto Rican	1.8 (0.9–2.8)	100.0 (100.0–100.0)
Other Hispanic	17.0 (13.7–20.3)	NA
Other race, non-Hispanic	5.8 (3.9–7.7)	NA

Abbreviation: NA, not applicable.

a The 14 states are Connecticut, Georgia, Indiana, Kentucky, Maryland, Michigan, Mississippi, Montana, Nebraska, New Jersey, Pennsylvania, Texas, Utah, Washington.

b Relative standard error of the estimate is 30%–50% (estimate is unreliable).

c Relative standard error of the estimate exceeds 50% and has been suppressed.

Across all races/ethnicities, at least 80% of respondents reported no smoking in the home, and 58% or more reported no pets in the bedroom (Table 2). The unadjusted analysis showed that use of a kitchen exhaust fan, use of a bathroom exhaust fan, avoiding pets in the bedroom, and washing linens in hot water varied by race/ethnicity (*P *< .01 for all).

**Table 2:** Prevalence of Indoor Environmental Control Practices, by Race/Ethnicity, Behavioral Risk Factor Surveillance System Child Asthma Call-Back Survey, 14 States^a^ and Puerto Rico, 2013–2014

Environmental Practice	14 States, No.	Total	NHW	NHB	Puerto Rican, Mainland	Other Hispanic	NHOR	Puerto Rican, Island (n = 60)
		% (95% Confidence Interval)						
Use of air purifier	1,404	30.3 (26.7–22.8)	25.2 (21.2–29.2)	36.8 (28.0–45.6)	44.8^b^ (18.4–71.3)	33.3 (22.2–44.3)	38.3 (21.6–54.9)	21.1 (8.7–33.4)
Use of dehumidifier	1,415	31.0 (27.4–34.6)	28.2 (23.6–32.8)	40.8 (31.5–50.2)	38.8^b^ (13.0–64.5)	25.9 (16.4–35.5)	31.5 (15.9–47.0)	30.1 (15.8–44.5)
Use of kitchen exhaust fan	1,406	70.9 (67.2–74.6)	68.5 (63.6–73.5)	71.9 (63.0–80.8)	73.2 (52.7–93.7)	79.3 (71.4–87.1)	63.2 (46.6–79.8)	34.6 (20.7–48.5)
Use of bathroom exhaust fan	1,399	60.4 (55.9–64.8)	64.4 (58.8–70.0)	54.4 (44.4–64.5)	62.9 (38.9–87.0)	55.3 (44.5–66.1)	59.8 (43.9–75.7)	15.5^b^ (6.2–24.9)
Use of dust mite–impermeable pillow cover	1,401	36.4 (32.4–40.4)	40.2 (34.7–45.6)	30.3 (21.8–38.7)	46.2 (19.3–73.1)	33.9 (22.6–45.3)	28.6^b^ (11.7–45.5)	53.7 (38.8–68.6)
Use of mattress encasement	1,389	30.3 (26.7–33.8)	48.8 (42.9–54.7)	45.8 (35.5–56.0)	49.2 (22.8–75.6)	36.9 (25.7–48.2)	37.7 (20.7–54.7)	60.3 (45.6–75.0)
No pets in bedroom^c^	1,418	67.6 (63.5–71.7)	58.3 (52.8–63.7)	87.9 (81.9–94.0)	80.2 (56.3–100.0)	66.5 (55.1–77.8)	75.9 (64.3–87.5)	82.1 (71.7–92.5)
No smoking in home	1,416	93.3 (91.4–95.1)	94.3 (92.0–96.7)	90.2 (85.3–95.1)	80.3 (58.9–100.0)	94.6 (90.5–98.7)	95.0 (93.3–96.7)	94.5 (89.6–99.4)
Linens washed in hot water^c^	1,397	41.8 (37.4–46.2)	41.3 (35.3–47.3)	32.4 (23.3–41.4)	39.6^b^ (13.2–66.0)	53.4 (42.5–64.3)	48.9 (31.8–66.1)	21.2^b^ (8.1–34.3)

Abbreviations: NHB, non-Hispanic black; NHOR, non-Hispanic other race; NHW, non-Hispanic white.

a The 14 states are Connecticut, Georgia, Indiana, Kentucky, Maryland, Michigan, Mississippi, Montana, Nebraska, New Jersey, Pennsylvania, Texas, Utah, Washington.

b Relative standard error of the estimate is 30%–50% (estimate is unreliable).

c Varies significantly by race/ethnicity in the 14 states per the Rao-Scott χ^2^ test.

The adjusted odds of using an air purifier, using a dehumidifier, and avoiding pets in the bedroom were higher for non-Hispanic black children than for non-Hispanic white children in the 14 states. For example, odds of restricting pets from the bedroom were approximately 4 times higher (aOR = 4.45; 95% confidence interval [CI], 2.26–8.75) for non-Hispanic black children than for non-Hispanic white children after adjusting for age, household income, and parental education level (Table 3). Households with Hispanic children had higher odds of using a kitchen exhaust fan (aOR = 1.79; 95% CI, 1.02–3.13) and washing linens in hot water (aOR = 1.81; 95% CI, 1.06–3.09), compared with non-Hispanic white children in the 14 states.

**Table 3:** Association Between Race/Ethnicity and Indoor Environmental Control Practices, BRFSS Child Asthma Call-Back Survey, 14 US States^a^ and Puerto Rico, 2013–2014

Environmental Practice	14 States, No.	NHW	NHB	Puerto Rican, Mainland	Other Hispanic	NHOR	Puerto Rican, Island^b^ (n = 60)
		Adjusted Odds Ratio (95% Confidence Interval)^c^					
Use of air purifier	1,404	1.00	2.00 (1.25–3.20)	3.4 (1.18–9.85)	1.55 (0.86–2.80)	1.66 (0.75–3.69)	0.73 (0.33–1.65)
Use of dehumidifier	1,415	1.00	1.84 (1.16–2.92)	1.68 (0.56–5.02)	0.88 (0.49–1.59)	1.20 (0.57–2.50)	1.02 (0.49–2.12)
Use of kitchen exhaust fan	1,406	1.00	1.25 (0.76–2.06)	1.14 (0.38–3.42)	1.79 (1.02–3.13)	0.81 (0.38–1.75)	0.22 (0.11–0.42)
Use of bathroom exhaust fan	1,399	1.00	0.75 (0.46–1.22)	1.02 (0.35–2.95)	0.93 (0.56–1.54)	0.86 (0.41–1.78)	0.14 (0.07–0.31)
Use of dust mite–impermeable pillow cover	1,401	1.00	0.89 (0.55–1.46)	1.96 (0.63–6.15)	1.17 (0.63–2.18)	0.50 (0.20–1.24)	3.59 (1.82–7.09)
Use of mattress encasement	1,389	1.00	1.11 (0.67–1.83)	1.31 (0.44–3.88)	0.87 (0.49–1.53)	0.59 (0.28–1.28)	2.44 (1.23–4.83)
No pets in bedroom	1,418	1.00	4.45 (2.26–8.75)	3.04 (0.68–13.67)	1.14 (0.60–2.16)	2.08 (1.06–4.09)	1.44 (0.68–3.04)
No smoking in home	1,416	1.00	1.03 (0.46–2.29)	0.36 (0.08–1.57)	2.43 (0.79–7.46)	1.29 (0.65–2.59)	1.69 (0.56–5.06)
Linens washed in hot water	1,397	1.00	0.76 (0.46–1.24)	1.08 (0.35–3.32)	1.81 (1.06–3.09)	1.27 (0.63–2.56)	0.44 (0.19–1.01)

Abbreviations: BRFSS, Behavioral Risk Factor Surveillance System; NHB, non-Hispanic black; NHOR, non-Hispanic other race; NHW, non-Hispanic white.

a The 14 states are Connecticut, Georgia, Indiana, Kentucky, Maryland, Michigan, Mississippi, Montana, Nebraska, New Jersey, Pennsylvania, Texas, Utah, Washington.

b Reference group includes children from the United States BRFSS Asthma Call-Back Survey data from 14 US states, 2013 and 2014.

c Odds ratios adjusted for age, household income, and education level.

The adjusted odds of using a kitchen exhaust fan (aOR = 0.22; 95% CI, 0.11–0.42) and odds of using a bathroom exhaust fan (aOR = 0.14; 95% CI, 0.07–0.31) were lower for children in Puerto Rico than for children in the 14 states. In contrast, the odds of using a dust mite–impermeable pillow cover (aOR = 3.59; 95% CI, 1.82–7.09) or mattress encasement (aOR = 2.44; 95% CI, 1.23–4.83) were highest among Puerto Rican children who are island residents. Although not significant, avoiding pets in the bedroom (aOR = 1.44; 95% CI, 0.68–3.04) was higher for children in Puerto Rico than for children in the 14 states.

## Discussion

The purpose of this study was to evaluate potential differences in IEC practices by race and ethnicity among children of vulnerable populations with asthma using data from the BRFSS ACBS. The study results indicate that these practices vary by race/ethnicity among children with asthma in 14 states and Puerto Rico. 

Our findings are mixed in comparison with a previously published study that investigated 4 states and did not include residents of Puerto Rico (10). The study found that non-Hispanic black children and Hispanic children were less likely to use mattress encasements and dust mite–impermeable pillow covers and were more likely to live in a home without pets compared to non-Hispanic white children (10). Our findings for pet avoidance by race/ethnicity are consistent with those of that study. In contrast, we did not observe a difference in the use of mattress and pillow covers by race/ethnicity in the 14 states. Furthermore, we observed higher odds for air purifier and dehumidifier use among non-Hispanic black children. National Health Interview Survey data from 2013 indicated that, compared with non-Hispanic white children, non-Hispanic black children also have a higher prevalence of receiving an asthma action plan, another NHLBI guideline recommendation (11).

A concerted effort is evident among families in Puerto Rico, who disproportionally experience asthma, to curb allergens that induce symptoms by using dust mite–impermeable pillow and mattress encasements, but these homes generally lack use of exhaust fans to clear irritants (mold and smoke) to children with asthma. Anecdotally, public health personnel familiar with Puerto Rico note that many homes do not have exhaust fans.

Our study has limitations. Our analysis was not designed to assess asthma control, which could potentially modify the association between race/ethnicity and implementation of IEC practices. Other limitations were the potential lack of generalizability to other US states/territories and the inability to evaluate the IEC practices in association with the medical management of asthma. The sample size was also limited, as evidenced by wide confidence intervals for the Puerto Rico sample and for Puerto Rican children in the continental states. 

Our study also has multiple strengths. Most notably, it used a more comprehensive population-based sample than did previous studies (6,10). In addition, previous literature has not looked at children in Puerto Rico separately, whereas we separately analyze this vulnerable group. Findings from this study can be used by public health and clinical providers to guide implementation of IEC practices for vulnerable races and ethnicities in platforms, including the Community Preventive Services Task Force Recommendation on Home-Based Multi-Trigger, Multicomponent Environmental Interventions for Children and Adolescents with Asthma (12), and the NHLBI Guidelines for the Diagnosis and Management of Asthma (13).

This population-based study indicated greater odds of implementing IEC practices among vulnerable racial/ethnic groups of children with asthma, compared with the less-affected non-Hispanic white population with asthma. Because there are differences in IEC practices among children of different racial/ethnic groups, health care providers and stakeholders developing public health guidelines could consider addressing an individualized intake and management plan for children. This customized approach would account for our study results, indicating where certain vulnerable populations successfully implement IEC practices and where other ethnic and racial groups are deficient. Additional evaluation is needed to assess the role of asthma severity, asthma control, and medical management in the association between race/ethnicity and implementation of IEC practices.
